# Landscape of T Cells in Tuberculous Pleural Effusion

**DOI:** 10.1111/crj.70066

**Published:** 2025-04-02

**Authors:** Lihui Zou, Jing Chen, Li Xie, Lili Zhang, Li Wan, Weimin Li, Hongtao Xu

**Affiliations:** ^1^ The Key Laboratory of Geriatrics, Beijing Institute of Geriatrics, Institute of Geriatric Medicine, Chinese Academy of Medical Sciences Beijing Hospital/National Center of Gerontology of National Health Commission Beijing China; ^2^ Beijing Economic and Technological Development Zone Annoroad Gene Technology Co., Ltd Beijing China; ^3^ Department of Tuberculosis Beijing Chest Hospital, Capital Medical University, Beijing Tuberculosis and Thoracic Tumor Research Institute Beijing China; ^4^ Clinical Biobank, Beijing Hospital, National Center of Gerontology, Institute of Geriatric Medicine, Chinese Academy of Medical Sciences Beijing China; ^5^ National Tuberculosis Clinical Lab of China Beijing Tuberculosis and Thoracic Tumor Research Institute, Beijing Chest Hospital, Capital Medical University Beijing China; ^6^ Department of Laboratory Medicine Beijing Hospital, National Center of Gerontology, Institute of Geriatric Medicine, Chinese Academy of Medical Sciences Beijing China

**Keywords:** CD4, CD8, single‐cell RNA sequencing, T cells, tuberculous pleural effusion

## Abstract

The distribution and the function of T lymphocyte subsets in pleural effusion (PE) and peripheral blood (PB) in tuberculous pleural effusion (TPE) patients remain unclear. In this study, we aimed to explore the expression patterns, regulatory mechanisms, and functions of T lymphocyte subsets in TPE patients, especially the distribution of T lymphocyte subsets at the single cell level. The CD3^+^ T cells were isolated from PE and PB of four TPE patients for single‐cell RNA sequencing (scRNA‐seq) to screen T cell subsets. T‐SNE projection, Gene Set Variation Analysis (GSVA), and pseudotime analysis were performed to analyze the composition, molecular and functional properties, and developmental trajectories of T cell subsets. Finally, ELISA was carried out to identify the cytokines secreted by PE and PB. We found that CD4^+^CD8^−^ T lymphocytes (Th1, Th2, and FOXP3^+^ Treg cells) were preferentially enriched in PE. The proportion of exhausted CD4^−^CD8^+^ cells in PE was higher than that in PB, while the proportion of initial and effector CD4^−^CD8^+^ cells was quite the reverse. We also found a large number of unexpected double positive (DP) cells in PE and PB, among which the proportion of CD4^+^CD8^+^‐C10‐CCL3 cells was the most different between PE and PB. Meanwhile, CD4^+^CD8^+^‐C10‐CCL3 was the group with the largest number of interactions with other groups. CD4^−^CD8^−^ cells were mainly found in PE and may be involved in the immunomodulatory effect of PE. Furthermore, the concentrations of cytokines secreted by Th1, Th2, and Treg in PE were higher than those in PB. Our study is helpful to understand the distribution pattern and dynamic changes of T cells in PE and PB of TPE patients and further understand that the functional status and regulation of T cells will be crucial for the successful development of TPE immunotherapy.

## Graphical abstract

This study explored the distribution and function of T‐lymphocyte subsets at the single cell level in TPE patients, and provided the scRNA‐seq analysis of T cells in the pleural blood compared to pleural effusions. We found the obvious heterogeneity of CD4^+^CD8^+^‐C10 and CD4^−^CD8^−^ cells in pleural effusion and pleural blood in TPE patients.

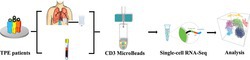

## 1 Introduction

Tuberculous pleural effusion (TPE), one of the most common forms of extrapulmonary tuberculosis, is characterized by pleurisy inflammation caused by *Mycobacterium tuberculosis* (MTB) infection [1, 2, 3, 4]. The clearance of MTB mainly depends on T cell‐mediated cellular immune response. Under the stimulation of MTB, sensitized T lymphocytes release various cytokines and recruit macrophages, which makes MTB more likely to be hydrolyzed, digested, and killed. Meanwhile, the inflammatory reaction caused by the activation of CD4^+^ and CD8^+^ T lymphocytes affects the capillary permeability and leads to the blockage of the lymphatic stomata in the parietal pleura, thus resulting in the imbalance between the formation and removal of pleural fluid [3, 5].

Most of the previous studies on TPE were based on the analysis of lymphocyte subsets with flow cytometry [6]. CD4^+^ T cells are mainly enriched in tuberculous pleurisy and have been classified into Type 1T helper (Th1) cells, Th2 cells, Th17 cells, Th9 cells, Th22 cells, and regulatory T cells (Treg cells) [7, 8]. The proportion and number of T lymphocyte subsets and immune balance will affect the pathophysiological process of related pleural lesions. For example, a high Th2 to Th1 ratio in pleural effusion (PE) was indicative of an immunocompromised status [9]. The imbalance of Th17/Treg exists in TPE, and pleural CD39^+^ Tregs inhibit the generation and differentiation of Th17 cells through a latency‐associated peptide‐dependent mechanism [10]. Some novel biomarkers and assays such as MTB DNA detection, ADA activity, and T‐STOP.TB assay have been explored for TPE diagnosis [11, 12, 13, 14]. However, there is still a lack of a high‐throughput and comprehensive interpretation of T cells in the tissues of TPE patients.

Single‐cell RNA sequencing (scRNA‐seq) provides a powerful tool for in‐depth analysis of immune cells. In principle, single‐cell immunome sequencing uses a combination of single‐cell isolation and sequencing. Individual immune cells are separated from complex cell populations by techniques such as micromanipulation or flow cytometry. These cells are then transcribed to detect their gene expression, thereby discovering differences and characteristics between different cells and then understanding the differentiation and function of immune cells. Several studies have obtained the global characteristics of T cells in human nonsmall cell lung cancer [15], breast cancer [16], or other types of tumor tissues and peripheral blood (PB) [17, 18]. The application of scRNA‐seq in various diseases provides strong evidence for the heterogeneity of gene expression between cells, the trajectory of cell subsets during development, and the discovery of rare cell types. However, there is only a small amount of single cell level analysis of MTB infection, especially TPE, which is highly related to T cell immunity. Moreover, little is known about the specific classification and function (cytokine secretion) of T cells in PE of TPE patients [19, 20, 21, 22].

In this study, we performed a scRNA‐seq of 6000 single T cells from each sample isolated from four TPE patients. The CD3 molecule is expressed on all human T cells and is an important marker for identifying T cells. Furthermore, according to the surface‐specific CD4 and CD8 molecules, T lymphocytes in peripheral blood can be divided into two main subgroups: CD4^+^CD8^−^ and CD4^−^CD8^+^ T cells. We identified CD4^+^CD8^−^ T lymphocytes (Th1, Th2, and Treg cells) that were dominant in PE; therefore, locally produced cytokines may be associated with the predominance of CD4^+^CD8^−^ T cells in PE of TPE patients. Meanwhile, we found that initial and effector CD4^−^CD8^+^ T cells were abundant and functionally active in PB of TPE patients. We also concerned that CD4^+^CD8^+^ cells were mainly distributed in PB and CD4^−^CD8^−^ cells accumulated in PE, which inhibit T cell's function by regulating proliferation and metabolism [23]. Although the function of CD4^−^CD8^−^ cells is still unclear, CD4^−^CD8^−^ cells enriched in PE indicated that the immune response was suppressed in the PE.

## 2 Materials and Methods

### 2.1 Patients

The diagnostic criteria of tuberculous exudative PEs were as follows: (1) symptoms of tuberculosis poisoning, (2) pulmonary tuberculosis focus, (3) exudative changes of PEs, (4) effective antituberculosis treatment for 2–4 weeks and rapid absorption of PEs, (5) MTB was found in sputum smear or sputum culture, (6) acid‐fast bacteria were found in PEs, and (7) typical tuberculous granuloma was found in pleura biopsies. All cases met the above 3 or more diagnostic criteria.

Inclusion criteria: (1) consistent with the diagnosis of tuberculosis PEs, (2) aged more than 14 years old and less than 70 years old, (3) hormone‐naive (never received hormone therapy before), and (4) antituberculosis treatment for less than 2 weeks.

Exclusion criteria: (1) complicated with autoimmune disease, (2) had undergone invasive thoracic surgery or had thoracic injury within 3 months before admission, and (3) complicated with PE caused by other causes.

### 2.2 Sample Collection and Single‐Cell Suspension Preparation

Specimens were collected from four patients with TPE within 24 h after admission. After the PE and PB of the above four patients with TPE were centrifuged, the cell precipitations were used for scRNA‐seq. Firstly, cells from PE and PB were centrifuged at 400 g for 5 min and then 10 X red blood cell lysis buffer (Solarbio, Beijing, China) was added to the cell precipitation of PB, which was incubated on ice for 10 min to remove red blood cells. The cell pellets of PE and PB were resuspended in Hank's buffer before the mononuclear cells were isolated using a Ficoll–Hypaque method (GE Healthcare Life Sciences, Milan, Italy). Then the cell pellet was resuspended in 80 μL of buffer and added 20 μL of CD3 MicroBeads (Miltenyi Biotec Inc., CA, USA) per 10^7^ total cells. After incubation for 15 min at 4°C, the cells underwent magnetic separation (Miltenyi Biotec Inc., CA, USA). Finally, the purity of the magnetically labeled cells was evaluated with flow cytometry.

### 2.3 ScRNA‐Seq Library Preparation and Sequencing

The single‐cell library was prepared with the 10 × Genomics Chromium single‐cell protocol using the v3 reagent kit (10 × Genomics, Pleasanton, CA, USA), according to the manufacturer's instructions. The cell suspension was loaded onto a chromium single‐cell chip together with the reverse transcription master mix. Reverse transcription was performed, which was followed by PCR amplification (98°C for 45 s, 13–18 cycles of 98°C for 20 s, 67°C for 30 s, 72°C for 1 min, and 72°C for 1 min). The amplified cDNA was then used for 5′ gene expression library construction. The single‐cell RNA library was sequenced on an Illumina NextSeq or HiSeq 4000 with a minimum sequencing depth of 25 000 reads/cell using the read lengths 26 bp Read1, 8 bp i7 Index, and 98 bp Read2.

### 2.4 ScRNA‐Seq Data Processing

For scRNA‐seq reads, cellranger count (10X Genomics, version 3.0.1) was used to compare scRNA‐seq reads to Homo_sapiens.GRCh38.91.chr reference genome. Before the data analysis, the samples were filtered for CD3^−^ cell population as well as the cells with low expression: The total expression of the detected genes was less than 500, and the proportion of mitochondrial genes was more than 20%.

The software for dimensionality reduction of cell populations was based on Seurat (R packet version 3.0.0). Principal component (PC) analysis was used to analyze 43 691 cell populations from 8 samples, and PCs with explanatory variance ranked in TOP20 were used for dimensionality reduction analysis. Clusters were identified using shared nearest neighbor (SNN) based on the first 20 PCs with *k* = 30 and resolution = 0.4. The same PCs were used to generate the t‐SNE projections, which were generated with a minimum distance of 1 and 20 neighbors.

### 2.5 Cell Proportion Comparison of Samples

The proportion of the cells was calculated as follows: Percent (clustern (Group)) = num (clustern (Group)) /num (Total (Group)).

Clustern (Group): the number of cells in the PE (or PB) sample in clustern; num (Total (Group)): the total number of cells in the PE (or PB) sample.

### 2.6 Gene Set Variation Analysis (GSVA) Analysis

GSVA (version 1.30.0) analysis was used to investigate the correlation between gene sets and phenotypes of samples. Eighteen pathways related to CD4^+^ T cells and 11 pathways related to CD8^+^ T cells (https://www.genome.jp/kegg/pathway.html) were selected for calculating the correlation between these pathways and each cell. When the difference between these two samples was analyzed, the limma was used to calculate the *p*‐value by grouping and comparing.

### 2.7 Pseudotime Analysis

Pseudotime analysis, also known as cell trajectory analysis, was performed by monocle2 (http://cole‐trapnell‐lab.github.io/monocle‐release/). The differentiation trajectory of cells or the evolution process of cell subtypes was determined mainly based on the expression patterns of key genes. Based on the sequence of gene expression changes that each cell must go through, individual cells were classified in pseudo‐time to simulate the dynamic changes in the process of time development.

### 2.8 Enzyme‐Linked Immunosorbent Assay (ELISA)

After the cell precipitations of the PE and PB of the above four patients were used for scRNA‐seq, the supernatants were collected for the detection of IFN‐γ, IL‐2, TNF‐a, IL‐4, IL‐5, IL‐10, and TGF‐β by ELISA according to the manufacturer's protocol (Cloud‐clone Corp., Wuhan, China). The diluent of the sample was added to the 96‐well plate and incubated at 37°C for 1 h. Subsequently, the detection reagent, substrate solution, and stop solution were added in turn, which were fully mixed and incubated at 37°C. Measurements were immediately carried out at 450 nm using an Epoch™ microplate spectrophotometer (BioTek, Winooski, VT, USA), and the concentration was calculated based on the OD value of the sample.

### 2.9 Statistical Analysis

Statistical analysis was performed in R (version 3.4.0) and GraphPad Prism 6.0 (GraphPad Software, CA, USA). Unpaired two‐sided Wilcoxon rank sum tests were used for pair‐wise comparisons. The Student *t* tests were performed for analyzing two groups of normally distributed data. The threshold of significance was defined as *p* < 0.05.

## 3 Results

### 3.1 Characterization of CD4^+^ and CD8^+^ T Cells Derived From scRNA‐Seq Data

**FIGURE 1:**
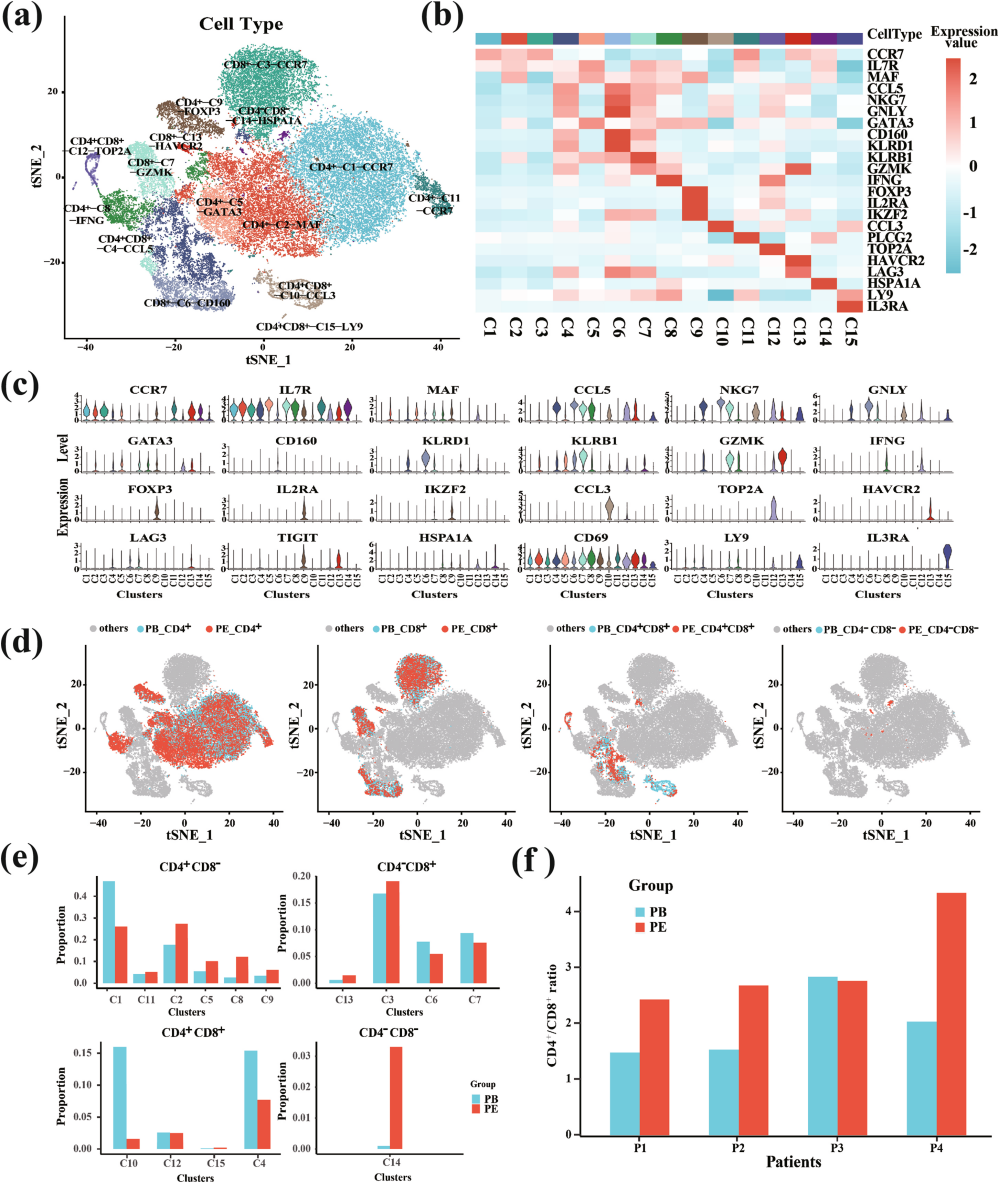
ScRNA‐seq of purified CD3^+^ single T cells from human PE and PB. (a) T‐SNE visually displayed 15 cell groups with a total of 43 691 cells from 8 samples, including 17 695 cells from PE samples and 25 996 cells from PB samples. (b) Heat map of the average expression of marker genes of T cells in each cluster. (c) Violin diagram of the average expression of marker genes of T cells in each cluster. (d) The t‐SNE plot of CD4^+^CD8^−^, CD4^−^CD8^+^, CD4^+^CD8^+^, and CD4^−^CD8^−^ cells in PE and PB samples. PE samples are shown in red, PB samples are shown in blue, and other types of cells are shown in gray. (e) Cell proportion distribution of four types of T cells in two different tissue samples. (f) Comparison of CD4^+^/CD8^+^ ratio in 8 samples of 4 TPE patients.

The basic information of the 4 TPE patients is summarized in Table 1. About 6000 cells per sample were sequenced by scRNA‐seq (the influence of doublets was excluded). To analyze the specific composition of T cells, we applied unsupervised clustering based on t‐SNE and densityClust (Figure S1). We found that in a total of 15 cell clusters, among which 6 clusters were CD4^+^ cell groups, 4 clusters were CD8^+^ cell groups, 4 clusters were CD4^+^CD8^+^ cell groups, and 1 cluster was CD4^−^CD8^−^ cell group (Figure 1a). According to the highly expressed genes of each cluster and the marker genes of T cell subclasses (Table S1), we identified these cell clusters as CD4^+^‐C1‐CCR7, CD4^+^‐C2‐MAF and so on (Table 2). Figure 1b demonstrated that the expressions of marker genes in similar clusters were relatively consistent. For example, the expression levels of *CCR7*, *IL7R*, and other genes in naïve cell groups (C1, C3, and C11) were relatively high, while the expression levels of cytotoxic genes such as *GNLY*, *NKG7*, and *GZMK* were very low. The violin distribution of marker genes in each cluster also showed that most of the marker genes were highly expressed only in their corresponding cluster (Figure 1c). For instance, *FOXP3*, a well‐known marker gene in Treg cells, was detected only in cluster 9 but not in other clusters.

**TABLE 1:** Basic information of TPE patients.

Patient	Age	Sex	Course	Therapy
1	43	Male	1 mouth	Antibacterial treatment
2	29	Female	12 days	None
3	16	Male	10 days	None
4	38	Female	2 weeks	None

*Note:* Course: time since diagnosis.

**TABLE 2:** Name of each cluster.

Cell type	Cluster ID	Classification	Cluster name	Marker gene
CD4^+^	C1	Naïve	CD4^+^‐C1‐CCR7	CCR7, LEF1, SELL
CD4^+^	C2	Th2	CD4^+^‐C2‐MAF	IL7R, FOS, JUNB, MAF
CD8^+^	C3	Naïve	CD8^+^‐C3‐CCR7	CCR7, LEF1
CD4^+^CD8^+^	C4	Effector	CD4^+^CD8^+^‐C4‐CCL5	CCL5, NKG7, GNLY, GZMA
CD4^+^	C5	Th2	CD4^+^‐C5‐GATA3	IL7R, GATA3, FOS, JUN, MAF
CD8^+^	C6	NKT	CD8^+^‐C6‐CD160	CD160, KLRD1
CD8^+^	C7	Cytotoxicity	CD8^+^‐C7‐GZMK	GZMK, KLRB1, CTLA2A
CD4^+^	C8	Th1	CD4^+^‐C8‐IFNG	CD74, GZMA, IFNG, JUN
CD4^+^	C9	Treg	CD4^+^‐C9‐FOXP3	FOXP3, IKZF2, IL2RA
CD4^+^CD8^+^	C10	NA	CD4^+^CD8^+^‐C10‐CCL3	S100A8, S100A9
CD4^+^	C11	Naïve	CD4^+^‐C11‐CCR7	CCR7, LEF1, PLCG2
CD4^+^CD8^+^	C12	Effector	CD4^+^CD8^+^‐C12‐TOP2A	—
CD8^+^	C13	Exhausted	CD8^+^‐C13‐HAVCR2	TIGIT, LAG3, HAVCR2
CD4^−^CD8^−^	C14	NA	CD4^−^CD8^−^‐C14‐HSPA1A	CD27, CD69
CD4^+^CD8^+^	C15	NA	CD4^+^CD8^+^‐C15‐LY9	IL3RA, LY9

According to the gene expression distribution of *CD4*, *CD8A*, and *CD8B*, all cell clusters were divided into four types: CD4^+^CD8^−^, CD4^−^CD8^+^, CD4^+^CD8^+^, and CD4^−^CD8^−^. Figure 1d demonstrated that CD4^+^CD8^−^ cell groups accounted for the largest proportion of all types of cells. From the distribution of CD4^+^CD8^−^, the proportion of naïve cells (C1) in PB was higher than that in PE samples, while the proportions of other types of CD4^+^CD8^−^ cell groups in PE samples were higher than those in PB. For CD4^−^CD8^+^ cell clusters, except for exhausted cells (C13), the proportions of the cells of other clusters in PB were higher than those in PE samples. The proportions of cells in PB were higher than those in PE samples in the distribution of most of the CD4^+^CD8^+^ cell clusters, but CD4^−^CD8^−^ cells were gathered in the PE samples (Figure 1e). CD4^−^CD8^−^ cells were reported to inhibit the proliferation of homologous CD4^+^CD8^+^ cells, which was consistent with the treatment of clinical fluid extraction [24]. The percentage and cluster proportion of cells in these two tissues of all samples represented the same conclusion (Figures S2 and S3).

The CD4^+^/CD8^+^ ratio of PE was significantly higher than that of PB, especially in patient 4, which further proved the aggregation of CD4^+^CD8^−^ cells in PE (Figure 1f). The CD4^+^/CD8^+^ ratio is an important indicator of the severity and prognosis of the disease [25]. The first patient, who had the lowest CD4^+^/CD8^+^ ratio, did not have any underlying immunosuppressive disease or had not used hormones before. However, he was treated with levofloxacin and cefoxitin before admission. Whether antibiotic treatment is related to the decrease of CD4^+^/CD8^+^ ratio, and whether there are other reasons, such as a mixed infection affecting CD4^+^/CD8^+^ ratio needs to be further explored.

### 3.2 Difference of CD4^+^CD8^−^ Cells Between PE and PB Samples

**FIGURE 2:**
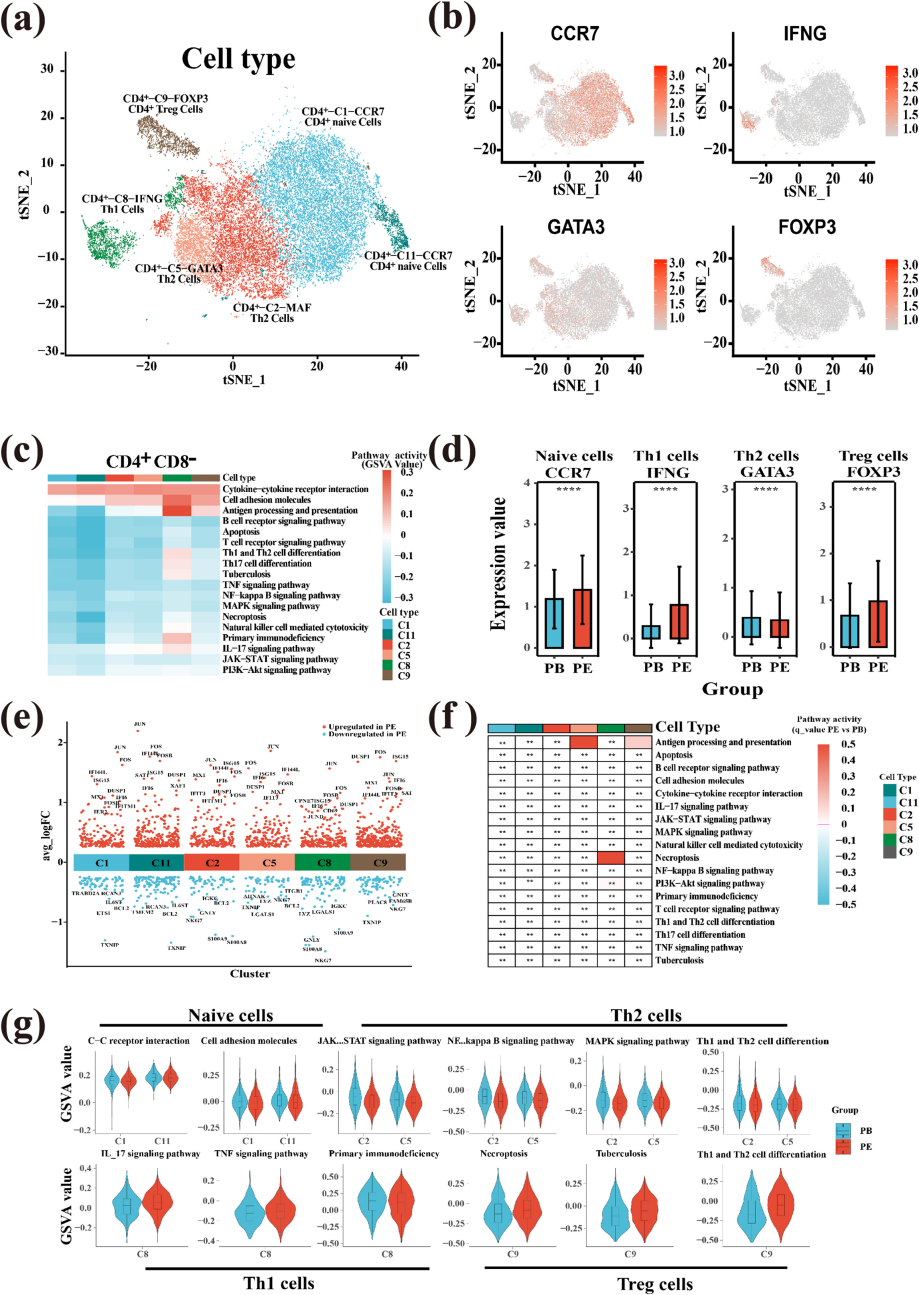
CD4^+^CD8^−^ subtype analysis based on single‐cell gene expression. (a) The t‐SNE projection of 6 main clusters shown in different colors. Cluster CD4^+^‐C1‐CCR7: naive CD4^+^ T cells; CD4^+^‐C2‐MAF: Th2 CD4^+^ T cells; CD4^+^‐C5‐GATA3: Th2 CD4^+^ T cells; CD4^+^‐C8‐IFNG: Th1 CD4^+^ T cells; CD4^+^‐C9‐FOXP3: Treg CD4^+^ T cells; CD4^+^‐C11‐CCR7: naive CD4^+^ T cells. (b) Distribution map of marker gene expression for the clusters as indicated. (c) Differences in pathway activities scored per cell by GSVA between the different clusters. (d) Analysis of the difference of marker gene expression between PE and PB in four types of CD4^+^ cell populations. The marked “****” means that the *p*‐value of the marker gene expression between PE and PB is less than 0.0001. (e) The distribution map of up‐ and down‐regulated genes in PE. The x axis represents cluster ID, and the y axis represents avg_logFC. Each dot represents a gene. The genes are upregulated in PE are shown in red, and the genes downregulated are shown in blue. (f) The GSVA significance test of the selected metabolic pathways carried out between the two tissues. The marked “**” means that the *q*‐value of the intercellular GSVA of the two tissues is less than 0.01, and the pathways marked in red indicate that there are no significant differences between the two tissues. (g) Distribution of GSVA value in two tissues in different CD4^+^ T cell populations. The *q*‐value of the intercellular GSVA of the two tissues is less than 0.01.s

A total of six CD4^+^CD8^−^ cell populations including naïve, Th1, Th2, and Treg cells (Figure 2a) were found based on the marker genes (Figure 2b). The pathway activities of Th1, Treg, and Th2 cell populations were higher than those of naïve cell group. In addition, the Th1 cell group had higher activity in tuberculosis, antigen presentation pathway, and Th1 and Th2 cell differentiation compared with other cell groups, so the immune response of TPE was mainly mediated by CD4^+^CD8^−^ Th1 cells (Figure 2c).

The expression levels of marker genes in PE were higher than that in PB in almost all types of CD4^+^CD8^−^ cell groups (Figure 2d). The inflammatory genes (*IFI44L*, *IFI6*, *IFIT3*, etc.) were mainly upregulated in PE, further indicating that the immune effect of PE in CD4^+^CD8^−^ cells was higher than that in PB (Figure 2e). The GSVA significance test demonstrated the functional differences between these two tissues in each cell group were significant, except for the three reddish pathways (Figure 2f). The pathways associated with Th1 and Treg were higher in PE than those in PB, especially the tuberculosis, autophagy, and Th1 and Th2 cell differentiation signaling pathways (Figure 2g). Thus, the proportions and activities of Th1, Th2, and Treg cell clusters in PE were the main factors that determine the function of cellular immune response, which were consistent with the results of previous studies.

### 3.3 Difference of CD4^−^CD8^+^ Cells Between PE and PB Samples

**FIGURE 3:**
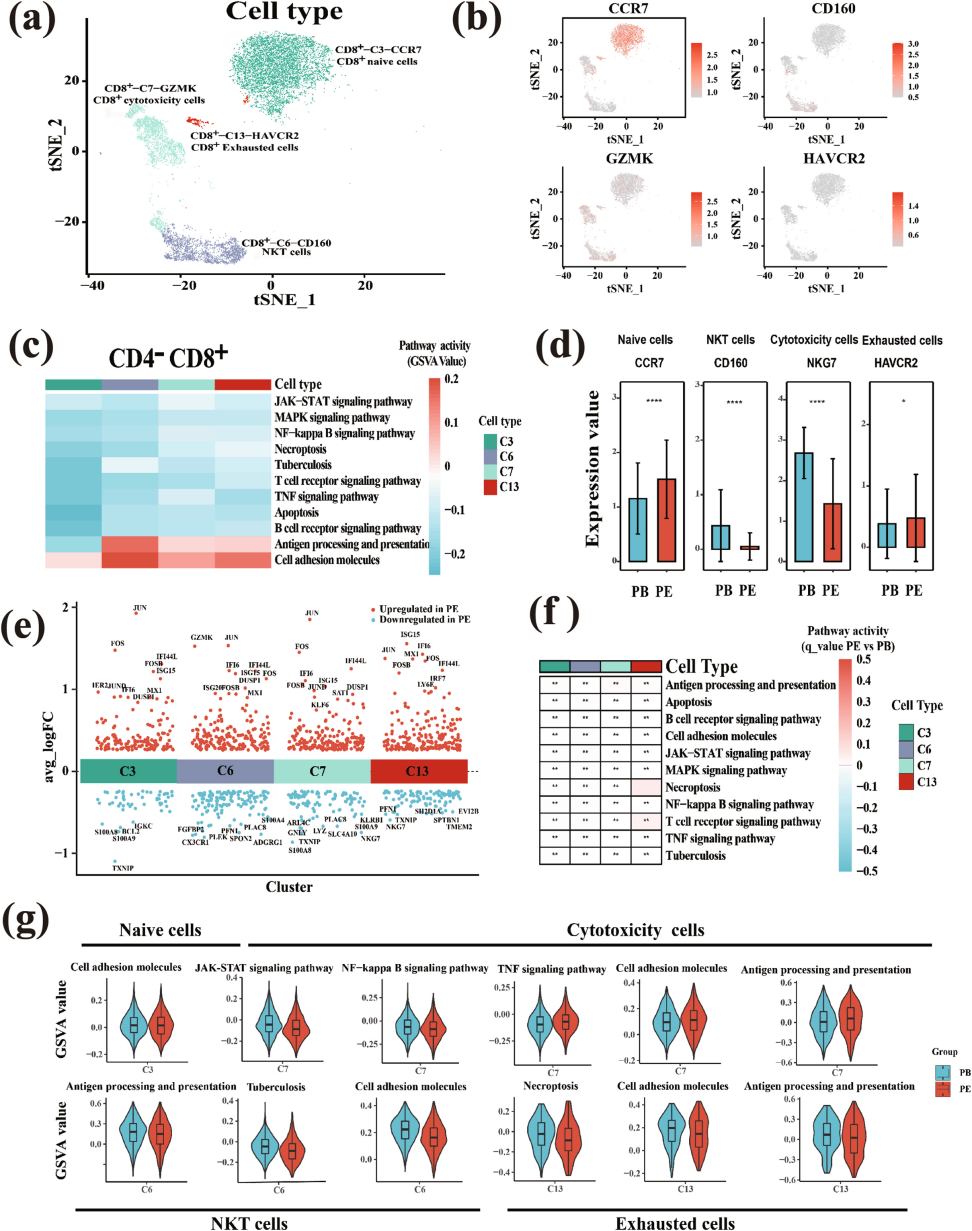
Characterization of CD4^−^CD8^+^ subtypes using single‐cell gene expression data. (a) The t‐SNE projection of 4 main clusters shown in different colors. Cluster CD8^+^‐C3‐CCR7: naive CD8^+^ T cells; CD8^+^‐C6‐CD160: NKT cells; CD8^+^‐C7‐GZMK: cytotoxicity CD8^+^ T cells; CD8^+^‐C13‐HAVCR: exhausted CD8^+^ T cells. (b) tSNE plot, color‐coded for expression (grey to red) of marker genes for the clusters as indicated. (c) Activities of pathways among different clusters as estimated using GSVA. (d) Average expression of marker genes from four types of CD8^+^ cell populations in PE and PB. The marked “*” and “****” mean that the *p*‐value of the marker gene expression between PE and PB is less than 0.05 and 0.0001, respectively. (e) The distribution map of dys‐regulated genes in PE compared with PB. The x axis represents cluster ID, and the y axis represents avg_logFC. Each dot represents a gene. The genes are upregulated in PE are shown in red, and the genes downregulated are shown in blue. (f) The GSVA significance test of the selected metabolic pathways carried out between the two tissues. The marked “**” means that the *q*‐value value of the intercellular GSVA of the two tissues is less than 0.01, and the pathways marked in red indicate that there are no significant differences between the two tissues. (g) Distribution of GSVA value in two tissues in different CD8^+^ T cell populations. The *q*‐value of the intercellular GSVA of the two tissues is less than 0.01.

For CD4^−^CD8^+^ cells, the four CD4^−^CD8^+^ cell clusters could be regarded as naïve, NKT, cytotoxicity, and exhausted cell groups (Figure 3a) according to the marker genes (Figure 3b). CD4^−^CD8^+^ cell populations were highly correlated with cell adhesion molecules and antigen processing and presentation pathways. Tuberculosis had the highest score in NKT cell group, indicating that the main cell group related to the tuberculosis pathway was NKT cell group (Figure 3c). The marker gene expression levels of NKT and cytotoxicity cells were significantly higher in PB than those in PE (Figure 3d), and the expression levels of cytotoxic genes such as *NKG7* and *GNLY* in PB were higher than those in PE (Figure 3e), indicating that the lethality of CD4^−^CD8^+^ cells in PE was lower than that in PB. However, the expression levels of marker genes of exhausted cells in PE were higher than those in PB, indicating that PE had stronger immune dormancy (Figure 3d).

For NKT cell group, the pathways of tuberculosis, antigen processing and presentation, and cell adhesion molecules, which were highly related to TPE, were higher in PB than those in PE (Figure 3f,g). All results demonstrated that in the CD4^−^CD8^+^ cell group, the immune reaction to tuberculosis infection of PE was weaker than that of PB.

### 3.4 Analysis of the Difference of DP Cell Cluster C10 in PE and PB Samples

**FIGURE 4:**
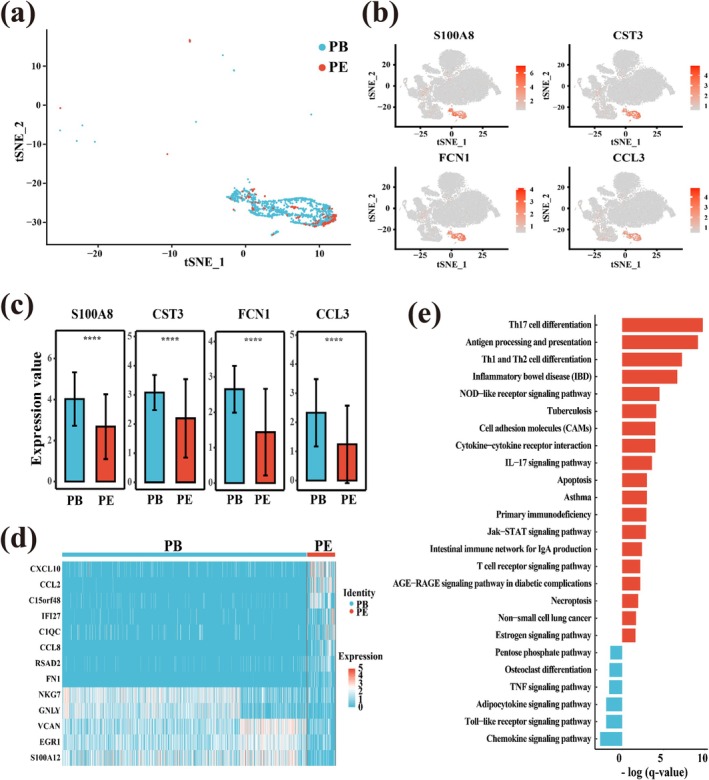
Detailed characterization of cluster 10 corresponding to CD4^+^CD8^+^ T cells. (a) The t‐SNE projection of the distribution of C10 in PB and PE. (b) The expression of marker genes of *S100A8*, *CST3*, *FCN1,* and *CCL3* in Cluster 10. (c) Average expression levels of marker genes of *S100A8*, *CST3*, *FCN1,* and *CCL3* from C10 in PE and PB. The marked “****” means that the *p*‐value of the marker gene expression between PE and PB is less than 0.0001. (d) *z*‐score normalized mean expression of selected C10‐associated genes in PE and PB. (e) The functional difference of differentially expressed genes of C10 in PE. The pathway values of differentially expressed genes in PE were shown by ‐log (*q*‐value). The red bars represent upregulated genes, and the blue bars represent downregulated genes.

We found that C10 had the greatest difference between the two tissues among the DP cell clusters. In PB and PE, there was a significant difference in C10 amount (the percentage of cells was 0.047 and 0.008, respectively) (Figure 4a). This group of cells mainly expressed non‐T cell type marker genes such as *S100A8*, *CST3*, *FCN1*, and *CCL3* (Figure 4b), and the expression levels of these genes in PB were higher than those in PE (Figure 4c). Furthermore, the expression levels of leukocyte migration‐related cytokines (*CCL2* and *CCL8*) were high in PE but very low in PB, which may be related to the need for cytokines to migrate to the site of infection (pleura). However, the expression levels of the cytotoxic genes *NKG7* and *GNLY* in PE were relatively low (Figure 4d). The functional difference analysis of up‐regulated and down‐regulated genes of C10 in PE showed C10 was highly correlated with Th17 cell differentiation pathway and antigen processing and presentation pathway, indicating that C10 affected TPE through many immune‐related pathways (Figure 4e). At last, the analysis of cell–cell interactions showed that C10 was the group with the largest number of interactions (Figure S4).

### 3.5 Relationship Among CD4^+^CD8^−^ T Cell Clusters Based on Developmental Trajectory

**FIGURE 5:**
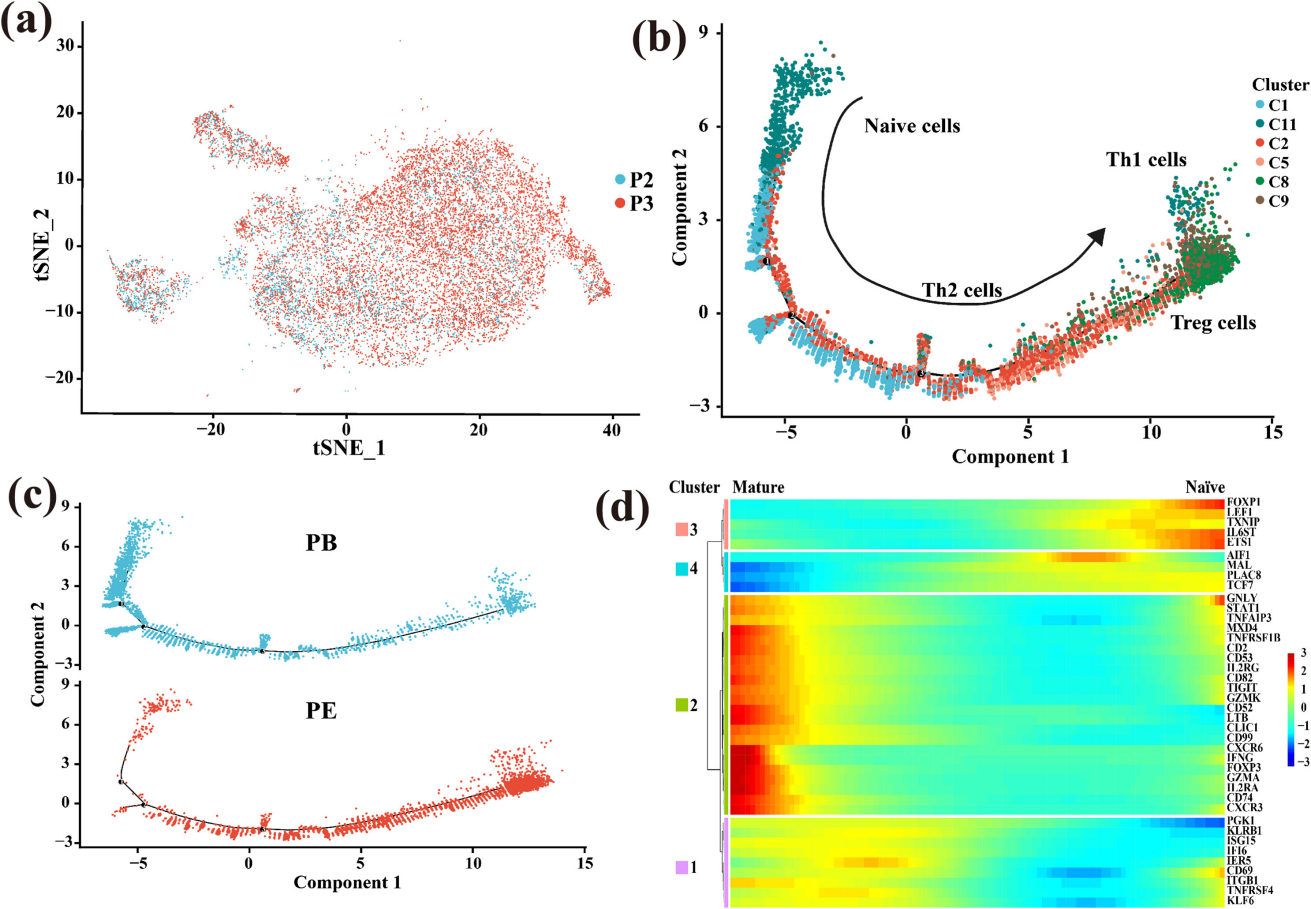
The development trajectory map of CD4^+^CD8^−^ cells. (a) Distribution of CD4^+^CD8^−^ T cell population in P2 and P3 samples. (b) The branching trajectories of CD4^+^CD8^−^ T cells in two‐dimensional space calculated by Monocle (version 2). Each point corresponds to a cell, and different clusters are distinguished by different colors. The arrow indicates the increasing direction of the state of CD4^+^CD8^−^ T cells. (c) The distribution map of PE and PB samples on the developmental trajectory. PE samples are shown in red, and PB samples are shown in blue. (d) Heat map of the expression of genes that determine the differentiation fate of CD4^+^CD8^−^ T cells. The *x*‐axis represents the cells arranged with the increase of the pseudo‐time value, the *y*‐axis represents the expression of the corresponding gene, and the pseudo‐time value is opposite to the direction of differentiation.

P1 had been treated with antibiotics, and P4 had a particularly high proportion of CD4^+^CD8^−^ cells and showed significant individual heterogeneity; thus, we used the sequencing data of P2 and P3 patients for developmental trajectory analysis (Figure 5a). With the increase of component 1, CD4^+^CD8^−^ T cells gradually developed from naïve state to Th2, Th1, and Treg states (Figures 5b). In the developmental trajectory, the proportion of naïve T cells was higher in PB than that in PE. With the progress of development, the proportions of Th1 and Treg cells were higher in PE than those in PB (Figure 5c). In addition, we selected the genes that best determine the differentiation fate of CD4^+^CD8^−^ T cells and were expressed by heatmap. With the development of differentiation, the function of naïve cells (*LEF1*, *IL6ST*) was getting weaker, but the function of Treg cell (*FOXP3*) population became stronger (Figure 5d).

### 3.6 Relationship Among CD4^−^CD8^+^ T Cell Clusters Based on Developmental Trajectory

**FIGURE 6:**
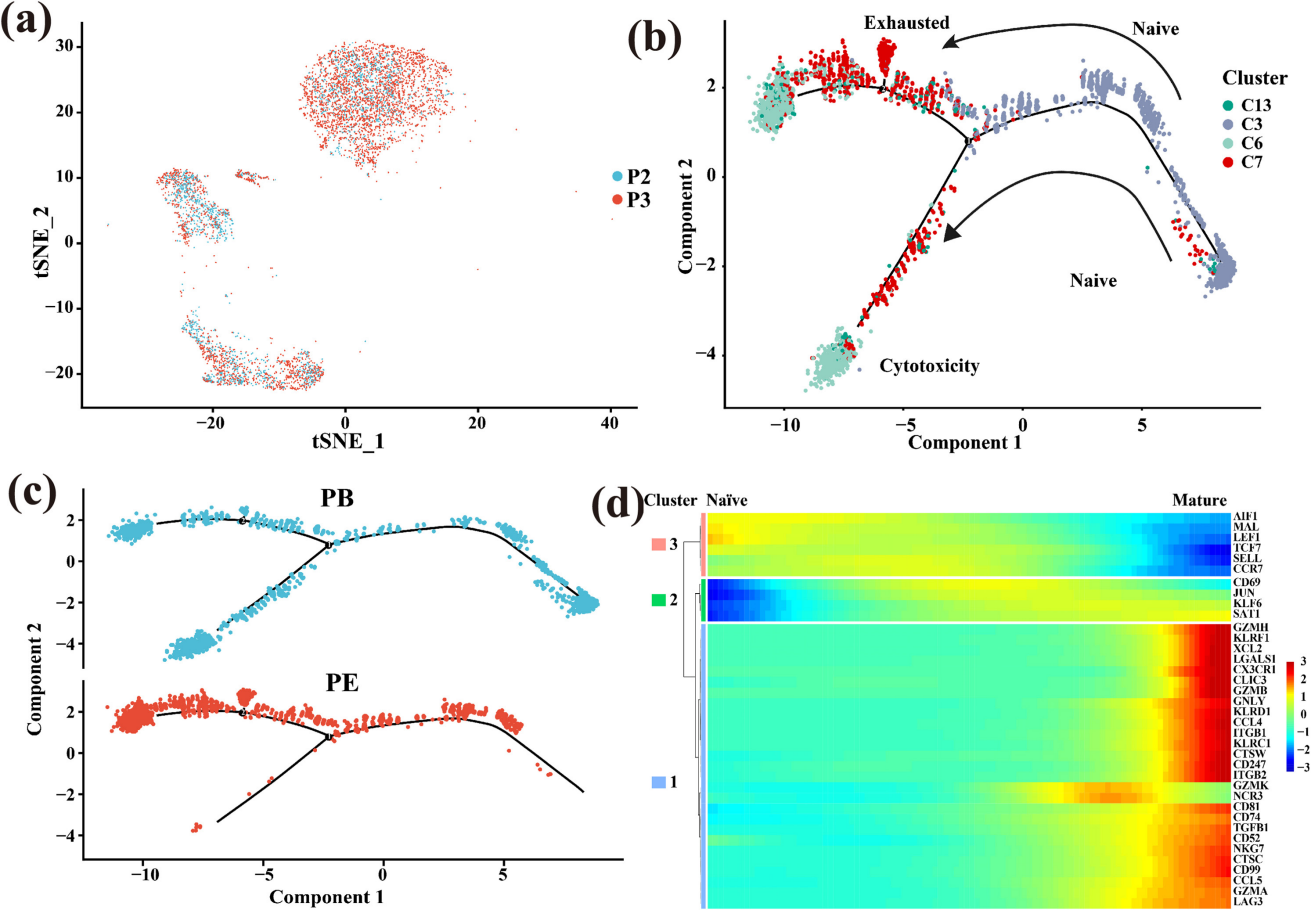
The development trajectory map of CD4^−^CD8^+^ cells. (a) Distribution of CD4^−^CD8^+^ T cell population in P2 and P3 samples. (b) The branching trajectories of CD4^−^CD8^+^ T cells in two‐dimensional space calculated by Monocle (version 2). Each point corresponds to a cell, and different clusters are distinguished by different colors. The arrow indicates the increasing direction of the state of CD4^−^CD8^+^ T cells. (c) The distribution map of PE and PB samples on the developmental trajectory. PE samples are shown in red, and PB samples are shown in blue. (d) Heat map of the expression of genes that determine the differentiation fate of CD4^−^CD8^+^ T cells. The *x*‐axis represents the cells arranged with the increase of the pseudo‐time value, the *y*‐axis represents the expression of the corresponding gene.

We also used the data of P2 and P3 patients for developmental trajectory analysis of CD4^−^CD8^+^ cell populations (Figure 6a). Naïve cells differentiated into two branches, C7 cytotoxicity cluster and C13 exhausted cluster (Figure 6b). Cytotoxicity T cells and exhausted T cells have two different cell fates in persistent chronic infection. Exhausted T cells lose the effector function of cytotoxicity T cells, which is characterized by the weakening of the ability to produce cytokines and the decreased capacity of cytotoxicity and cell proliferation. In the later stage of development, the PE samples were mainly differentiated into exhausted cells, while the PB samples were mainly differentiated into cytotoxicity cells (Figure 6c). The expression heatmap also demonstrated that the function of naïve (*CCR7*) was getting weaker, but the functions of cytotoxicity (*GZMH*, *GNLY*) and exhausted (*KLF6*) cell population were getting stronger with the differentiation (Figure 6d).

### 3.7 ELISA Findings of Cytokines Secreted by PE and PB

**FIGURE 7:**
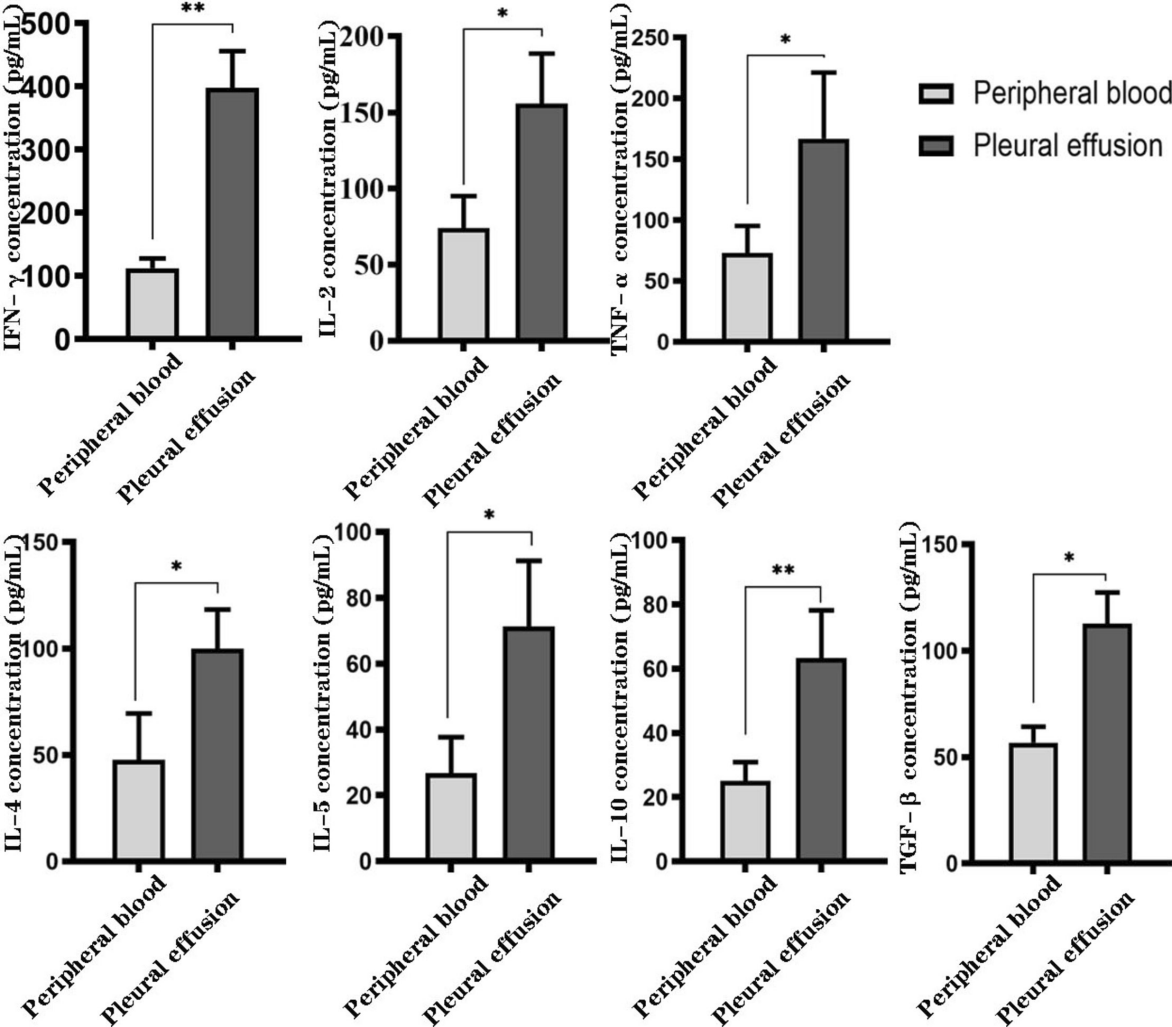
Relative expression level of IFN‐r, IL‐2, TNF‐a, IL‐4, IL‐5, IL‐10, and TGF‐β in PE and PB in four patients. Concentrations of IFN‐γ, IL‐2, TNF‐a, IL‐4, IL‐5, IL‐10, and TGF‐β in PE and PB supernatant of four patients were measured by ELISA. Data were represented as means ± SD. * *p* < 0.05; ** *p* < 0.01 (Student *t*‐test).

Because CD4^+^CD8^−^ T cells were dominant lymphocytes present in PE, we determined the expression levels of Th1‐secreted cytokines (IFN‐γ, IL‐2, and TNF‐α), Th2‐secrected cytokines (IL‐4, IL‐5, and IL‐10) and Treg‐secrected cytokines (TGF‐β) in PE and PB of the above 4 patients with TPE. We observed that PE expressed higher levels of IFN‐γ, IL‐2, TNF‐α, IL‐4, IL‐5, IL‐10, and TGF‐β than PB (Figure 7). Our results were also consistent with those of previous studies with larger sample size. Previous results showed that the median concentrations of IFN‐γ, IL‐2, IL‐4, and IL‐10 in PE of TPE were 823.37, 43.76, 26, and 38.69 ng/L respectively, each being statistically higher than that in the serum (118.60, 0.00, 1.49, and 0.00 ng/L, respectively) [26]. There were higher levels of TNF‐α (25.43 ± 13.55 pg/mL) and IFN‐γ (114.97 ± 27.85 ng/L) in tuberculous PE than nontuberculous PE [27, 28].

## 4 Discussion

By using scRNA‐seq, we found that CD4^+^CD8^−^ and CD4^−^CD8^+^ single positive cells, especially CD4^+^CD8^−^ cells, accounted for the largest proportion in the pathogenesis of TPE. In addition, we analyzed the function of T lymphocytes and the developmental trajectory of T cells. Many studies have demonstrated that TPE is a T helper cell disease dominated by Th1 [7, 29, 30], which was further confirmed in our current study. Besides, we observed that PE expressed higher concentrations of Th1 cytokines (IFN‐γ, IL‐2, and TNF‐α) than PB, which can promote the excessive inflammatory reaction and increase pleural adhesion, pleural thickening, and local pleural tissue damage caused by tuberculosis infection [31]. Meanwhile, our current study found that the proportion and the extent of activation (cytokine secretion) of Th2 cells in PE were higher than those in PB. Therefore, the role of Th2 cells in TPE cannot be ignored.

MTB cannot be completely removed from tuberculosis patients, leading to persistent infection. The mechanism of escaping immune surveillance is closely related to Treg cells [32, 33]. Compared with PB, Treg accounted for a significantly increased percentage of total PE cells, which can reduce excessive inflammation and lead to immune surveillance avoidance [34, 35]. The number of CD4^+^ FOXP3^+^ T cells was associated with pleural fluid adenosine deaminase (ADA) levels in TPE [36], which act as an early diagnostic indicator of the disease [37]. As a transcription factor, FOXP3 protein can affect the activity of NFAT and NF‐κB, resulting in the inhibition of IL‐2, IL‐4, and IFN‐γ production [38]. Therefore, the increase in the number of TPE Treg cells and the accompanying inhibition of cytokine production may lead to the imbalance between immunity and tolerance, but the specific mechanism needs to be further studied.

There have been few previous studies on CD4^−^CD8^+^ cells and TPE, and only a few studies mentioned that both naive and memory CD4^−^CD8^+^ T cells can be observed in recent tuberculosis infections [39, 40], although neither one predominates over the other. Generally speaking, CD4^−^CD8^+^ naive T cells can differentiate into CD4^−^CD8^+^ effector T cells in peripheral lymphoid organs after being stimulated by antigen in acute infection. Whether the antigen is cleared or not, some effector T cells persist to form memory T cells with the ability of self‐renewal to produce a rapid immune response to re‐infection [41]. On the contrary, the differentiation of naive T cells will be different under the persistent chronic infection such as MTB, and T cells will differentiate into exhausted T cells [42]. Our study demonstrated that the proportion of exhausted CD4^−^CD8^+^ cells in PE was higher than that in PB, which was also consistent with the fact that chronic infection with MTB in PE can lead to dysfunction or exhaustion of effector T cells in the immune system. Therefore, T cell exhaustion is a common feature of TPE and will become a major target in immunotherapy. In addition, we found the marker gene expression levels of NKT cells were significantly higher in PB than those in PE. These CD8^+^ NKT cells can function as antigen‐specific suppressive cells to regulate the immune response through killing antigen‐bearing DCs [43], so as to prevent immune overreaction and immune injury.

A small number of DP and double negative (DN) cells has been identified in autoimmune and chronic inflammatory disorders, but their function is still controversial [44, 45, 46, 47]. In our current study, the amount of DP cell cluster C10 cells was different in PE and PB. According to the surface markers of these DP cells we found, they did not express or downexpress the surface markers of immature T cells (such as *TdT*, *CD7* ^*dim*^) but express non‐T cell type marker genes such as *S100A8*, *CST3*, and *CCL3*. We speculate that these DP cells are generated from single positive cells during the infection. Similar to the position preference of C10 cells, the expressions of its marker genes such as *S100A8*, *CST3*, *FCN1,* and *CCL3* in PB were much higher than those in PE. Previous studies have found that S100A8 and MIP‐1alpha (CCL3) were remarkably elevated in PE of tuberculosis patients [48, 49, 50]. Both S100A8 and CCL3 play a key role in regulating the inflammatory response by stimulating the recruitment of proinflammatory cells. The difference in the distribution of C10 in PE and PB may be related to the cell migration after pathogen infection, but the specific mechanism needs to be further studied. Considering that more than 95% of the PB of healthy are CD4 or CD8 single positive T cells [51], the positive results obtained in this study provide us with confidence that C10 has the potential to be a biomarker in the PB of TPE. We also found that CD4^−^CD8^−^ T cells were mainly present in PE, and its proportion was much higher in PE than that in PB. The CD4^−^CD8^−^ T cells regulate the immune response as a double‐edged sword. It has been reported that DN T cells (gamma delta T cells) play a pro‐inflammatory role in TB [46]. However, there are also reports in the literature about the human DN Treg cell that directly contributes to the protective host response against MTB infection [47]. Whether the DN cells found in this study play a pro‐inflammatory or immunosuppressive role in the pathogenesis of TPE, or dynamically regulate the stability of the immune system, needs to be further studied.

In conclusion, this preliminary study identified the distribution of T lymphocyte subsets at the single cell level in TPE patients. However, the inevitable limitations of our study were that the sample size was small (4 patients) and no comparison with relevant controls, such as healthy individuals and non‐MTB infection‐induced pleural inflammation or some other non–infection induced pleural inflammation. The heterogeneity between PE and PB in TPE patients needs to be further verified in a large proportion of samples, and the PB of TPE patients also needs to be compared with healthy controls. In addition, we should not only characterize the immune composition of TPE with high resolution but also track their function dynamically. Finally, the roles of PE in the immune microenvironment of TPE were also worthy of further study.

## 5 Conclusions

In summary, CD4^+^CD8^−^ cells mainly play an immune role in PE, while CD4^−^CD8^+^ cells mainly act in PB. There is obvious heterogeneity between CD4^+^CD8^+^‐C10 and CD4^−^CD8^−^ cells in PE and PB in patients with TPE. Therefore, the detection of T lymphocyte subsets is expected to be a useful auxiliary test for the diagnosis of TPE. In addition, improving the immune function of T lymphocyte subsets is helpful to improve the therapeutic effect of TPE.

## Author Contributions

Hongtao Xu and Weimin Li contributed to the design of the project. Li Xie provided clinical samples and relevant information. Jing Chen and Lili Zhang performed sequencing and data analysis. Li Wan performed the validation of variations. Lihui Zou performed the experiments and data analysis and wrote the manuscript.

## Ethics Statement

The study was approved by the ethics committee of Beijing Hospital (2017BJYYEC‐108‐04), and all the subjects signed informed consent forms.

## Conflicts of Interest

Author Jing Chen was employed by Annoroad Gene Technology Co. The remaining authors declared that the research was conducted in the absence of any commercial or financial relationships that could be construed as a potential conflict of interest.

## Supporting information


**Figure S1** The distribution of all cell clusters in each sample.

**Figure S2** Percentage distribution of all cell clusters in PB and PE.

**Figure S3** The cluster frequency of all cell clusters in PE and PB of 4 patients.

**Figure S4** The cell–cell interactions of all cell clusters.

## Data Availability

The data that support the findings of this study are openly available in Genome Sequence Archive for Human at https://bigd.big.ac.cn/, reference number subHRA001267.
