# Supplementary figures and images for: Landscape of T Cells in Tuberculous Pleural Effusion
Source: Clin Respir J. 2025 Apr 2;19(4):e70066. doi: 10.1111/crj.70066 (PMC11962214; doi:10.1111/crj.70066)

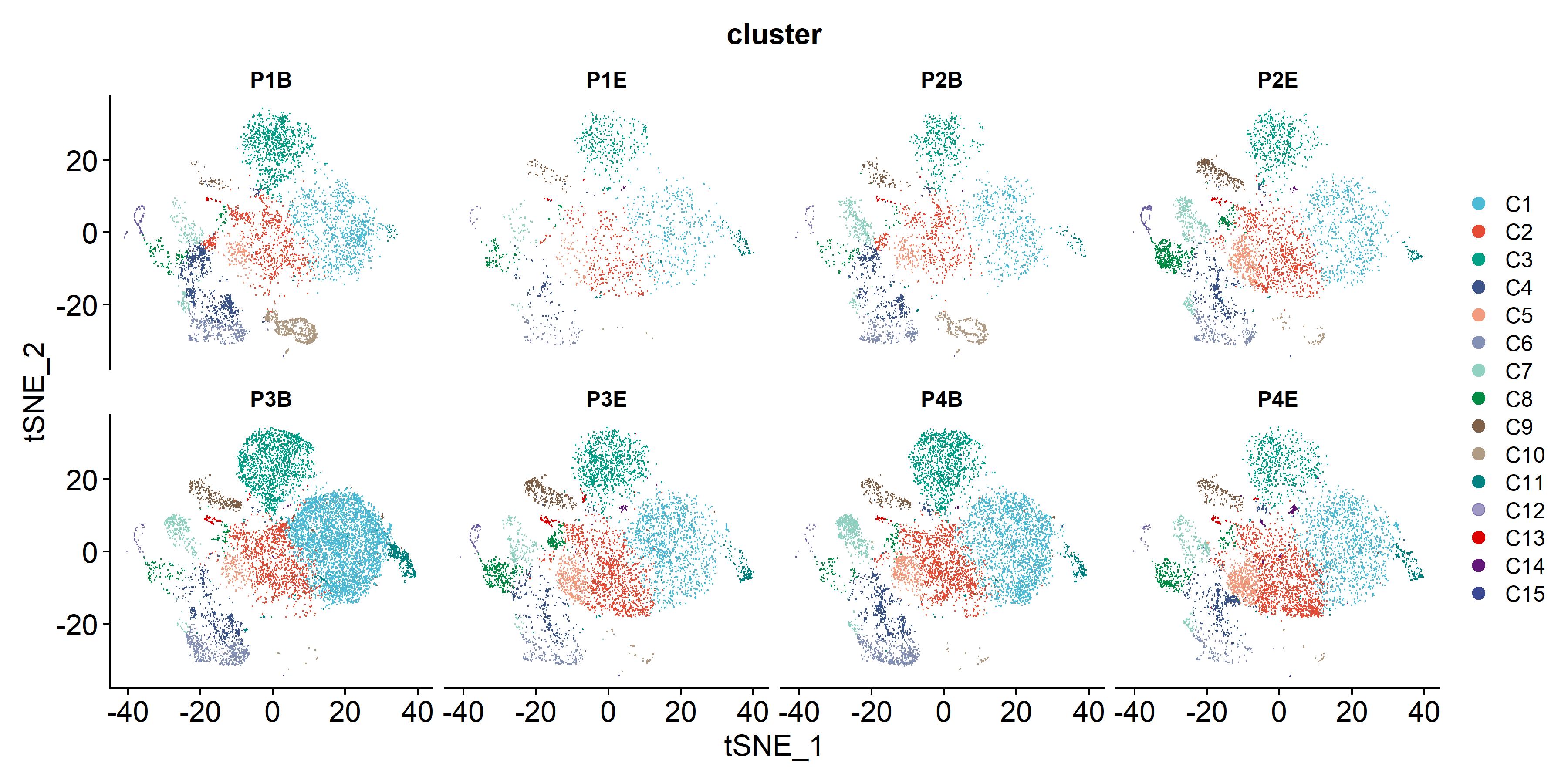

Supplement: Supplementary file 1 — Figure S1 The distribution of all cell clusters in each sample. [file CRJ-19-e70066-s004.jpg]

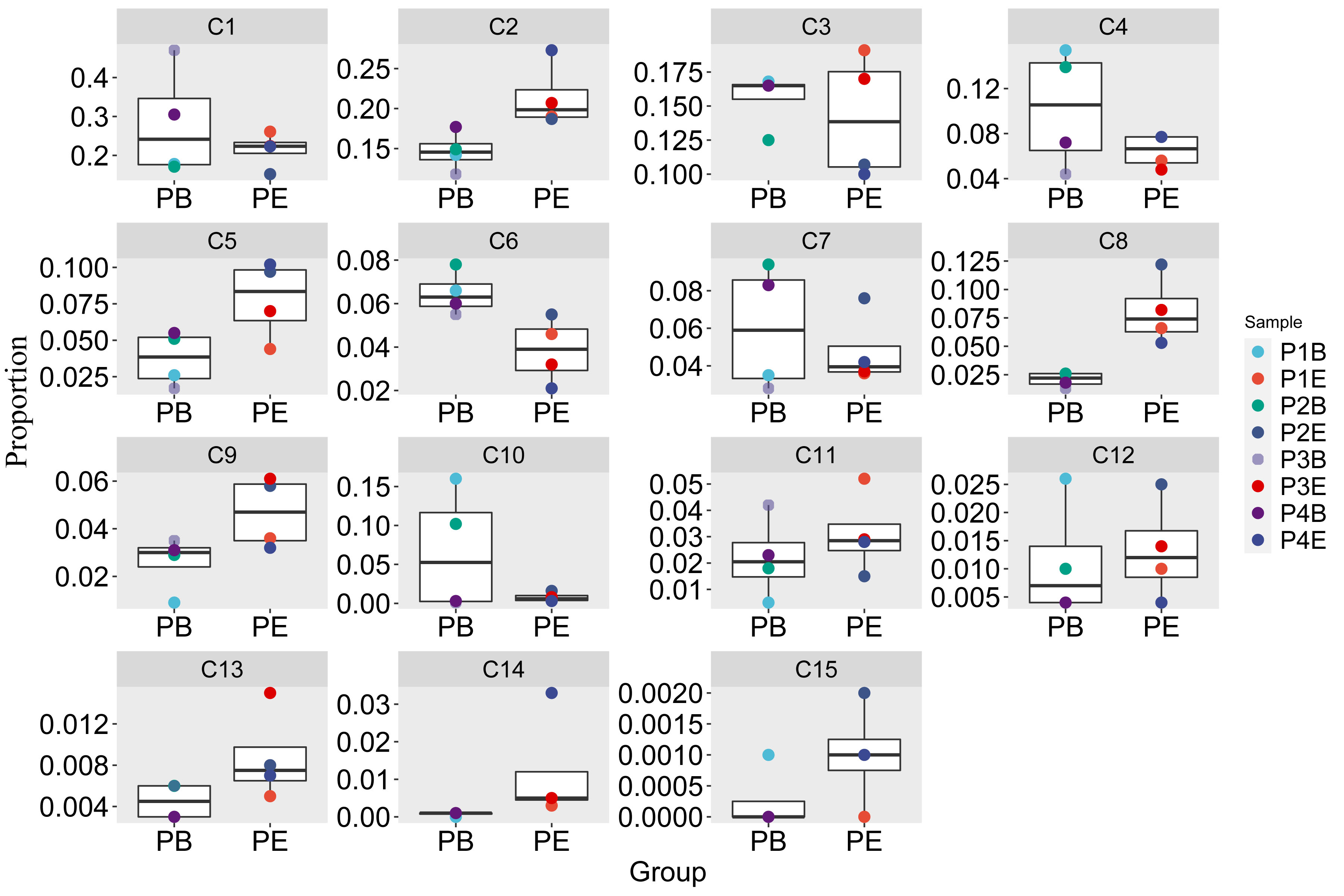

Supplement: Supplementary file 2 — Figure S2 Percentage distribution of all cell clusters in PB and PE. [file CRJ-19-e70066-s002.jpg]

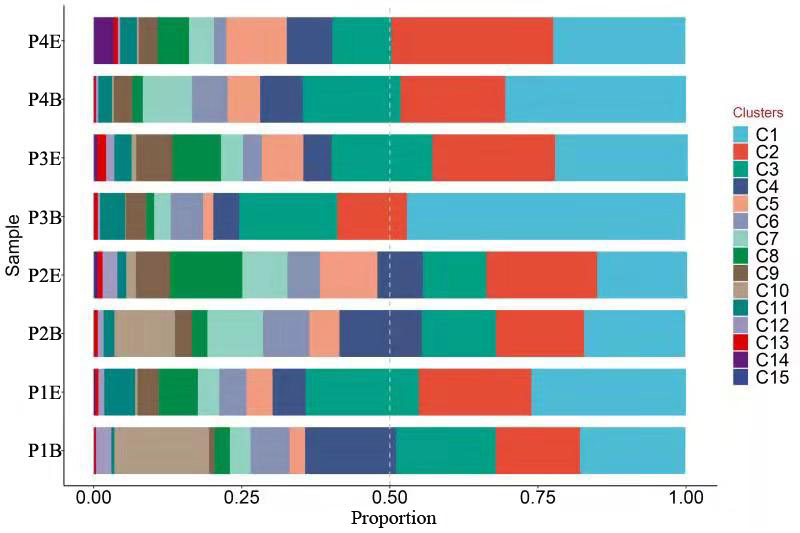

Supplement: Supplementary file 3 — Figure S3 The cluster frequency of all cell clusters in PE and PB of 4 patients. [file CRJ-19-e70066-s003.jpg]

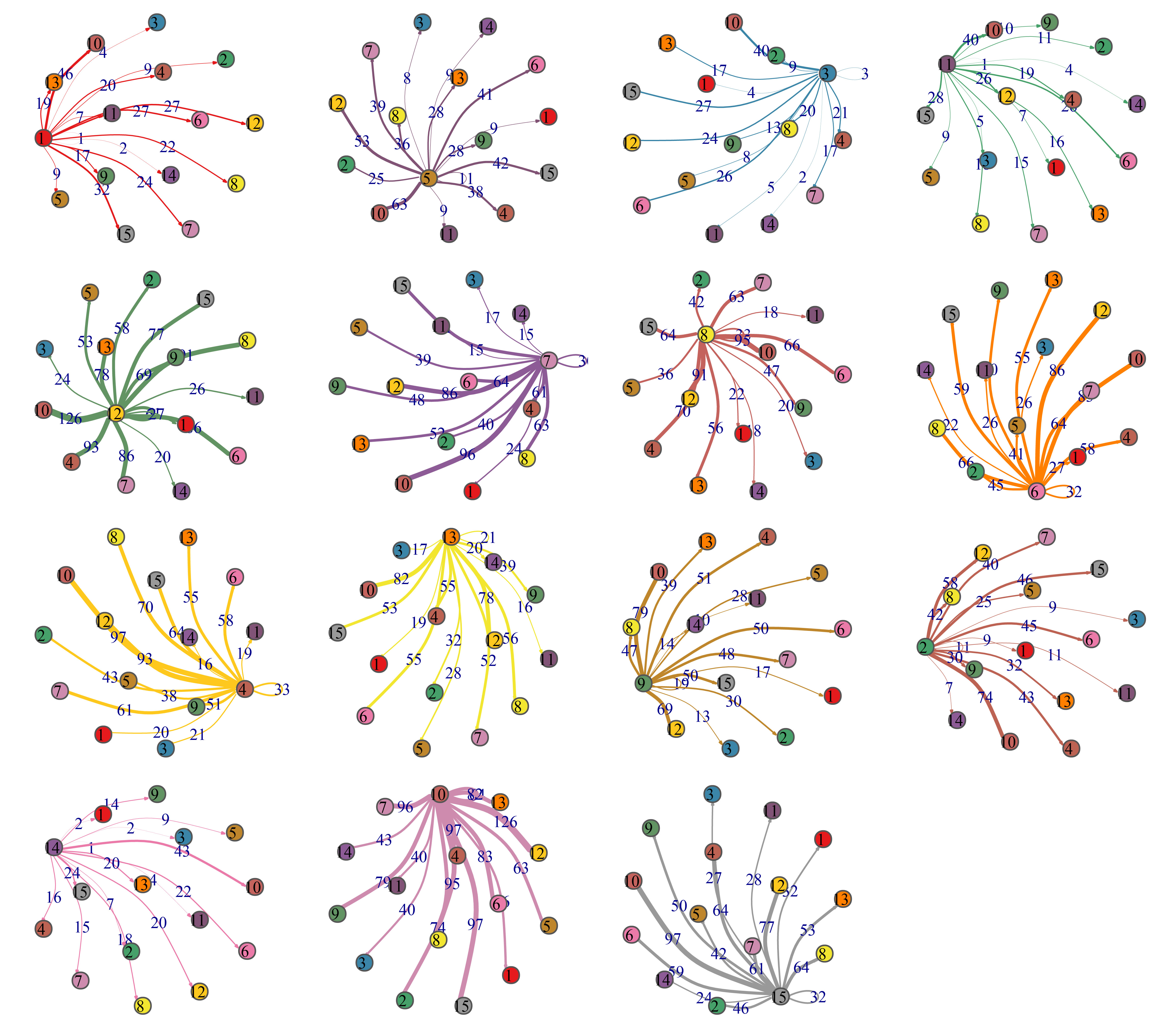

Supplement: Supplementary file 4 — Figure S4 The cell–cell interactions of all cell clusters. [file CRJ-19-e70066-s001.jpg]
